# MeioCapture: an efficient method for staging and isolation of meiocytes in the prophase I sub-stages of meiosis in wheat

**DOI:** 10.1186/s12870-018-1514-z

**Published:** 2018-11-21

**Authors:** Arun S. K. Shunmugam, Venkatesh Bollina, Stefanie Dukowic-Schulze, Pankaj K. Bhowmik, Chris Ambrose, James D. Higgins, Curtis Pozniak, Andrew G. Sharpe, Kevin Rozwadowski, Sateesh Kagale

**Affiliations:** 1 National Research Council Canada, Saskatoon, SK Canada; 2 Department of Horticultural Science, University of Minnesota, St. Paul, MN USA; 3 Department of Biology, University of Saskatchewan, Saskatoon, SK Canada; 4 Department of Genetics and Genome Biology, University of Leicester, Leicester, UK; 5 Department of Plant Sciences, University of Saskatchewan, Saskatoon, Canada; 6 Global Institute for Food Security, University of Saskatchewan, Saskatoon, Canada; 7 Agriculture and Agri-Food Canada, Saskatoon, SK Canada

**Keywords:** Meiosis, Meiocytes, Sporogenous archesporial column, Wheat, Cytogenetics, Meiotic prophase, Functional genomics

## Abstract

**Background:**

Molecular analysis of meiosis has been hindered by difficulties in isolating high purity subpopulations of sporogenous cells representing the succeeding stages of meiosis. Isolation of purified male meiocytes from defined meiotic stages is crucial in discovering meiosis specific genes and associated regulatory networks.

**Results:**

We describe an optimized method termed MeioCapture for simultaneous isolation of uncontaminated male meiocytes from wheat (*Triticum spp.*), specifically from the pre-meiotic G2 and the five sub-stages of meiotic prophase I. The MeioCapture protocol builds on the traditional anther squash technique and the capillary collection method, and involves extrusion of intact sporogenous archesporial columns (SACs) containing meiocytes. This improved method exploits the natural meiotic synchrony between anthers of the same floret, the correlation between the length of anthers and meiotic stage, and the occurrence of meiocytes in intact SACs largely free of somatic cells. The main advantage of MeioCapture, compared to previous methods, is that it allows simultaneous collection of meiocytes from different sub-stages of prophase I at a very high level of purity, through correlation of stages with anther sizes. A detailed description is provided for all steps, including the collection of tissue, isolation and size sorting of anthers, extrusion of intact SACs, and staging of meiocytes. Precautions for individual steps throughout the procedure are also provided to facilitate efficient isolation of pure meiocytes. The proof-of-concept was successfully established in wheat, and a light microscopic atlas of meiosis, encompassing all stages from pre-meiosis to telophase II, was developed.

**Conclusion:**

The MeioCapture method provides an essential technique to study the molecular basis of chromosome pairing and exchange of genetic information in wheat, leading to strategies for manipulating meiotic recombination frequencies. The method also provides a foundation for similar studies in other crop species.

**Electronic supplementary material:**

The online version of this article (10.1186/s12870-018-1514-z) contains supplementary material, which is available to authorized users.

## Background

Meiosis is a highly conserved process that is essential for fertility in sexually reproducing organisms. The process of meiosis occurs in specialized cells called meiocytes, and involves three principal events that include chromosome pairing, recombination and segregation [1]. Although the cytological events during meiosis are well characterized, the mechanisms controlling meiotic progression, chromosome recognition, pairing between homologous or homoeologous chromosomes and recombination are still poorly understood. In plants, polyploidy adds an extra layer of complexity to the meiotic process. Polyploid crops remarkably display diploid-like meiotic behavior and disomic inheritance despite having highly similar homoeologous chromosomes [2]. The underlying genetic control of strict homologous chromosome pairing in polyploid plants, such as commercial hexaploid bread wheat (*Triticum aestivum*, 2n = 6x = 42; AABBDD), tetraploid pasta wheat (*Triticum durum*, 2n = 4x = 28; AABB) or canola (*Brassica napus*, AACC) is not fully understood yet [3].

Prophase I is the longest (taking up to 90% of the total duration) and arguably most important phase of meiosis. It is divided into the five sub-stages leptotene, zygotene, pachytene, diplotene and diakinesis, during which a series of closely integrated and spatiotemporally controlled events occur, including condensation and reorganization of the chromosomes, pairing and synapsis of homologs, recombination and crossing over [4]. A comprehensive understanding of these processes requires a thorough understanding of their gene regulatory networks and catalytic and structural proteins. A prerequisite for the application of global genomic and proteomic profiling approaches to elucidate genetic interactions and pathways controlling meiosis is the availability of methods that allow isolation of high purity meiocytes from plant reproductive tissues. In case of plant female meiosis, collecting female meiocytes at a sufficient scale is currently untenable due to their inaccessibility and occurrence in a relatively lower number in the germline lineage [5, 6]. Although the male meiocytes are present in large numbers within the anther tissues, the complex morphological structure of the anther makes the isolation of male meiocytes also challenging.

**Fig. 1:**
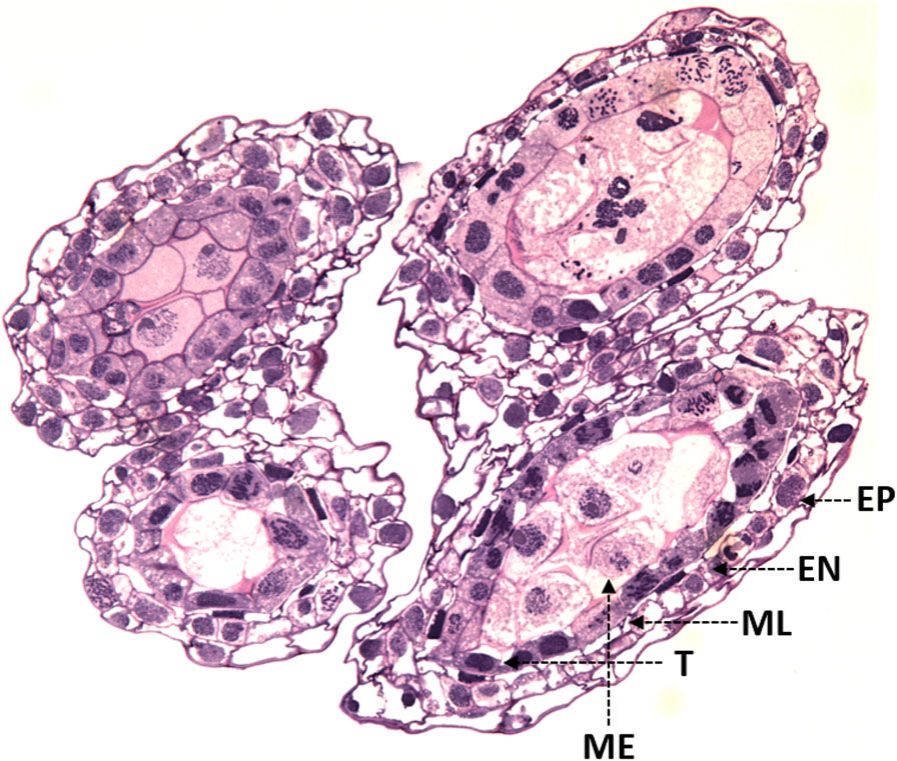
Semi-thin cross section of wheat anther stained with toluidine blue dye shows the anatomy of anther with four locules and multiple concentric layers, including epidermis (EP), endodermis (EN), middle layer (ML), tapetal layer (T) and meiocytes (ME), within each locule

A wheat inflorescence, also referred to as an ear, spike or head, consists of a main axis with several lateral spikelets. Each spikelet has at least three florets and each floret has an ovule and three stamens which produce pollen in terminal sac-like structures called anthers. The semi-thin cross section of an anther in Chinese Spring (CS) wheat shows the characteristic four-lobed structure (Fig. 1). Each lobe consists of central meiocytes [also referred to as pollen mother cells (PMCs) or microsporocytes] surrounded by non-meiotic cell layers comprising of outer epidermis, endothecium, middle layer and inner tapetum (Fig. 1). The floral meristem consists of three concentric histogenic layers, designated as L1, L2 and L3, which give rise to different anther tissues following stamen primordia initiation [7]. Except for the epidermis and the connective tissue, which arise from L1 and L3 layers of anther primordium, respectively, the remaining tissues originate from the L2 layer. Some of the L2 cells develop into archesporial cells which then divide into the sub-epidermal primary parietal layer and sporogenous cells or meiocytes [8]. The archesporial initials of meiocytes in each locule form an intact structure, the sporogenous archesporial column (SAC) within each locule of the wheat anther. The SACs are surrounded by a single layer of somatic tapetal cells which nourish the growing male gametes throughout their development. The tapetal cells undergo synchronous mitosis at the same time the pollen mother cells are undergoing meiosis [9]. Thus, use of an anther squash technique to isolate prophase I male meiocytes typically incurs contamination from tapetal and surrounding epidermal cells.

Several methods have been previously described or proposed for the isolation of male meiocytes [6], such as micromanipulation (*Plumbago* [10, 11], *Nicotiana* [12], *Brassica* [13, 14], *Arabidopsis* [15–17] and sunflower [18]), capillary collection of meiocytes (CCM) (*Arabidopsis* [19, 20] and maize [21, 22]), laser capture microdissection (LCM) in rice [23–26], Percoll gradient separation (*Arabidopsis* [27, 28], rice [29] and *Brassica* [30]) and isolation of nuclei tagged in specific cell types (INTACT) in *Arabidopsis* [31]. However, these methods have drawbacks that affect their use in the simultaneous isolation of large numbers of meiocytes from prophase I sub-stages for omics studies. For instance, micromanipulation, despite being a common technique practiced for the past three decades for isolation of male and female gametes from plants, involves enzymatic digestion of non-reproductive tissues surrounding the cells of interest combined with manual collection using a micromanipulator [32], and hence it is a time-consuming and inefficient method. CCM is a modified micromanipulation technique that involves the use of thin glass capillary tubes to isolate meiocytes under an inverted microscope. This is a successfully used method for isolating meiocytes [19–22], but it is also time-consuming. LCM involves fixing, embedding and sectioning of plant tissues that contain cells of interest under an expensive laser beam device connected to a microscope [33], which is again quite laborious. Unlike micromanipulation, CCM and LCM, Percoll gradient separation is a high-throughput method, but it requires high amounts of input material, and relies on the size and mass of the cells, which might not be sufficiently distinguishable when it comes to pre-meiotic and early prophase I cells [27–30]. INTACT is a transgenic method in which nuclei of the cell type of interest are tagged with biotin-labels and then affinity-purified from other tissues in the pool [31]. The need for transgene expression makes this method inconvenient and often unfeasible.

Here we describe MeioCapture, a rapid and highly reproducible technique to isolate intact SACs containing pre-meiotic nuclei or meiocytes from individual sub-stages of prophase I in wheat anthers. Meiocytes in anthers occur as a column of cells (SACs) largely free of somatic cells, and can be extruded using a dissection needle without any contamination [13]. The MeioCapture protocol exploits this anatomy along with the natural meiotic synchrony between anthers of the same floret, and the strong relationship between the size of wheat anther and the associated meiotic stage for collection of meiocytes from different stages simultaneously. This method is applicable to other crops and it is particularly useful in transcriptomic and proteomic studies where the purity of meiocytes is critical.

## Methods

### Plant material and growth conditions

Seeds of bread wheat (*Triticum aestivum* L.) genotypes CS and Fielder were obtained from Plant Gene Resources of Canada (http://pgrc3.agr.gc.ca/index_e.html). Stettler seeds were kindly provided by Agriculture and Agri-Food Canada (AAFC), Swift Current, SK, Canada. Plants were grown in the growth facility at National Research Council Canada, Saskatoon, SK, Canada. Four-inch pots filled with Sunshine® Mix #8/LC8 (Sun Gro Horticulture Canada Ltd., Seba Beach, AB, Canada) were used to grow plants in a controlled environment chamber (PGW40; Conviron, Winnipeg, MB, Canada) set at 21 ± 1 °C constant temperature with 16 h day length. Fluorescent lights (Sylvania®, LEDVANCE, Mississauga, ON, Canada) delivering a photosynthetic photon flux density (PPFD) of 400 μmol photons m^− 2^ s^− 1^ were used to illuminate the chamber. The plants were watered every day and fertilized every two weeks with water-soluble 20–20-20 fertilizer at the rate of 3.0 g/L and chelated micronutrient mix at the rate of 0.3 g/L (both from Plant Products Co. Ltd., Brampton, ON, Canada). Developing wheat inflorescence were collected eight weeks after seeding when the spikelets were found to be at the meiotic stages of interest. The developing inflorescences within the leaf sheath were checked for appropriate meiotic stages by gently sensing them with fingers. The spikes were collected from at least 20 plants for CS and 8 plants for Stettler and Fielder genotypes, placed in a beaker with distilled water on ice and transferred to the lab for meiocyte isolation. Only the spikelets from the primary inflorescence were harvested to maintain consistency in age and position of the spikelet used for meiocyte collection.

### Light microscopy

Developing wheat spikelets were excised under a dissecting microscope (Wild Leitz, Willowdale, ON, Canada) fitted with an ocular micrometer to measure anther lengths. Acetocarmine (2%) stained anthers were observed with a light microscope (OPTIKA B-290-TB, Optika®, Ponterancia, BG, Italy) to confirm the meiotic stages. The images of semi-thin cross sections of wheat anthers and the different meiotic stages were captured with a Leica DMR light microscope (Leica Microsystems, Wetzlar, Germany) attached to a MacroFire colour camera by Optronics (Scientific Instrument Company, Campbell, CA, USA). The whole anther images were photographed under bright field using a Zeiss Axio Zoom V16 stereo microscope (Carl Zeiss Microscopy GmbH, Jena, Germany). The images were organized and edited in Canvas X application (Canvas GFX, Inc., FL, USA) and Adobe Photoshop CS6 graphics editor (Adobe Systems, CA, USA).

### Transmission electron microscopy (TEM)

Transmission electron microscopy was performed using standard operating protocols for plant tissues. The size sorted anthers (0.5, 0.6, 0.7, 0.8, 0.9, 1.0, 1.1, 1.2 and 1.4 mm) from CS wheat genotype were fixed overnight with 2% glutaraldehyde. The fixed anthers were transferred to 1 M Sodium Cacodylate [(NaCac); pH 7.2 to 7.4] and fixed for 2 h at room temperature. The anthers were then osmicated in 1% osmium tetroxide (OsO4) for 1 h at room temperature. Osmicated samples were rinsed with double distilled water and dehydrated in a graded ethanol series (50, 70, 95, 100, 100 and 100%) for 10 min at room temperature at each grade. The samples were subsequently infiltrated at room temperature with LR White resin (London Resin Company, London, UK) as follows; 1 part LR White: 1 part 100% ethanol for 2 h, 2 parts LR White: 1 part 100% ethanol for 3 h and finally with pure LR white overnight. Infiltrated samples were blocked by placing them in 4 mm diameter TAAB embedding capsules (TAA Laboratories Equipment Limited, Berks, UK) and filling the capsules with fresh medium grade LR white resin. The samples were then polymerized overnight at 65 °C. Ultra-thin and semi-thin sections of the samples were generated using an Ultramicrotome Leica EM UC7 (Leica Microsystems, Germany). The sections were photographed with a Hitachi HT7700 digital transmission electron microscope (Hitachi High-Technologies Corporation, Tokyo, Japan).

### Confocal microscopy

The SACs were imaged using a Zeiss LSM 510 meta mounted on a Zeiss Axiovert 200 M inverted microscope with a 25X air objective (N.A. 0.8; Carl Zeiss, Oberkochen, Germany). The LSM510/ConfoCor2, version 3.2 SP2 software was used for image capturing from the microscope. The SACs were imaged and processed using the ImageJ image processing and analysis tool [34].

### The MeioCapture method

**Fig. 2:**
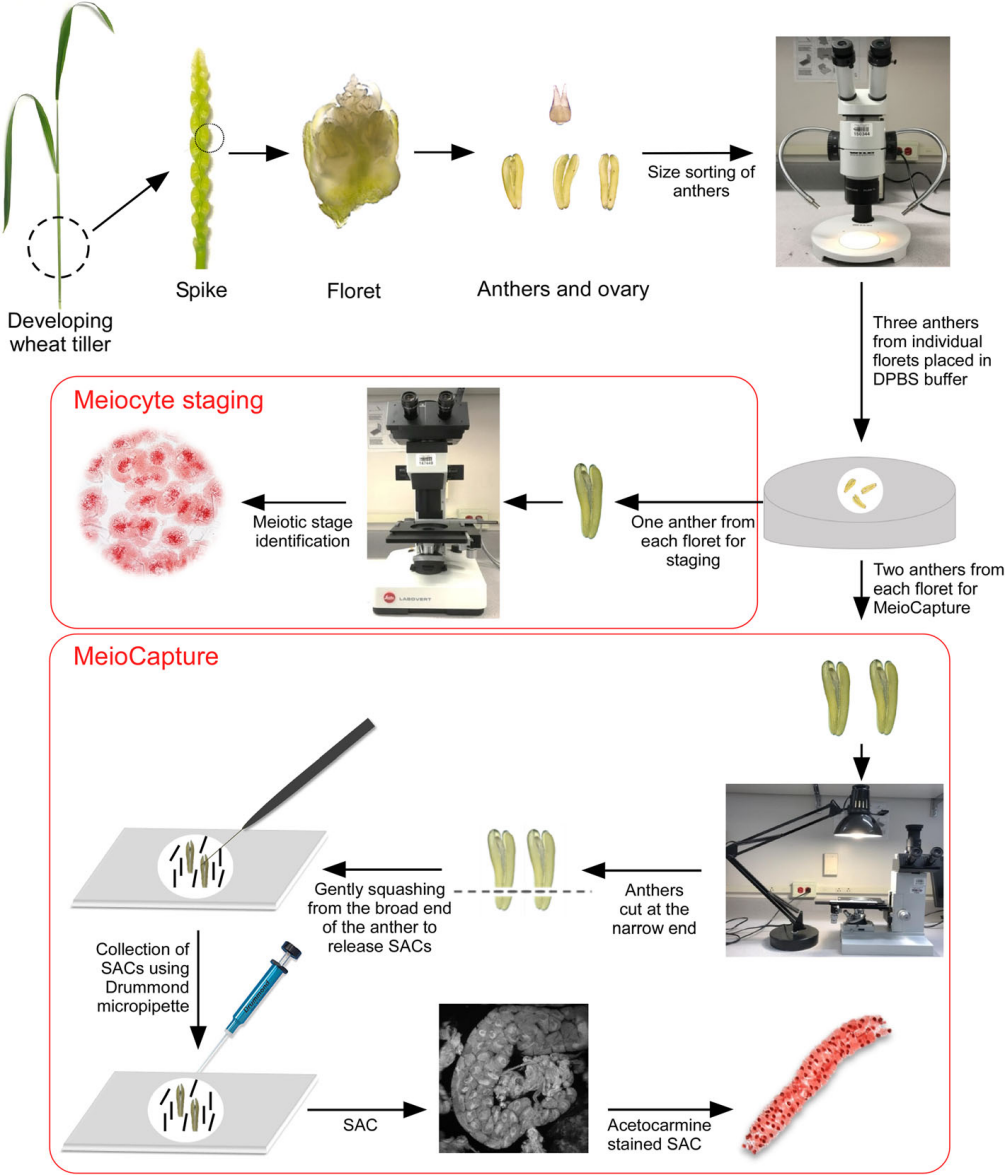
Schematic representation of MeioCapture technique for meiocyte isolation in wheat. A developing wheat tiller, young spike, floret, ovary and the anthers isolated from the floret are shown. For staging and meiocyte isolation, anthers are isolated and size sorted under a dissection microscope. After careful staging of one out of the three anthers from each floret, meiocytes are collected from pooled anthers by MeioCapture method. Anthers in DPBS buffer are cut at the narrow end, squeezed gently from the broad end to release the sporogenous archesporial columns (SACs) which are collected using a Drummond microdispenser

This section describes the whole procedure for isolation of SACs in detail and step-by-step. A schematic representation of the optimized method for isolation and staging of meiocytes is shown in Fig. 2. All the steps of the protocol can be carried out using standard laboratory equipment and microscopes. A summary of the duration of each meiotic stage, associated key events and the number of meiocytes isolated per hour using the MeioCapture method is provided in Table 1.

**Table 1:** Characteristics of meiotic prophase I and metaphase I stages in wheat and the efficiency of the MeioCapture protocol

Meiotic stage	Duration in CS (hour)^a^	Key events	No. of meiocytes isolated per hour, per person using the MeioCapture method^b^	Condition of SACs
Pre-meiotic G2	–	DNA replication and chromosome organization	500 to 700	Intact
Leptotene	10.4	Chromosome association	1000 to 1200	Intact
Zygotene	3.4	Homologue pairing and SC formation	1000 to 1200	Intact
Pachytene	2.2	Crossing over and bivalent formation	1400 to 1500	Intact
Diplotene	0.6	Dissociation of SCs	1500	Intact
Diakinesis	0.4	Nuclear membrane disintegration and end of prophase I	300 to 400	Starting to disintegrate
Metaphase I	1.6	Chromosomes line up on metaphase plate	700 to 800	Partially disintegrated

*CS* Chinese Spring, *SC* synaptonemal complex, *SAC* sporogenous archesporial column

^a^The duration of each meiotic stage provided is based on data from [9, 43, 44]

^b^ The total number of meiocytes isolated per hour varies depending on the mix of stages occurring in a spike

### Microscopes

1. A dissection microscope with an ocular micrometer
2. A light microscope with 5, 10 and 40X objectives (and 10X ocular magnification)
3. An inverted microscope with 5, 10 and 40X objectives (and 10X ocular magnification)

### Reagents

1. 1X Dulbecco’s phosphate-buffered saline (DPBS) buffer (ThermoFisher, Catalog no. 14190144)
2. RNase Away (ThermoFisher, Catalog no. 10328011)
3. RNA*later* (ThermoFisher, Catalog no. AM7020)
4. Trizol reagent (ThermoFisher, Catalog no. 15596018)
5. 2% acetocarmine staining solution (Ward’s Science, Catalog no. 470300–054)
6. 100% ethanol

### Tools for isolation and collection of meiocytes

1. Watchmakers forceps
2. Dissecting needles (self-made; see step 13 below)
3. Plastic coverslips (Fisherbrand, Catalog no. 12–547)
4. Plastic slides (Fisherbrand, Catalog no. S67112A)
5. Glass slides
6. 5 μL Drummond fixed volume microdispenser (Drummond, Catalog no. 3–000-105)
7. 1 to 5 μL volume Drummond capillary tubes (Drummond, Catalog no. 3–000-105-G)
8. 60 × 5 mm plastic Petri dish (Fisherbrand, Catalog no. FB0875713A)
9. 150 × 21 mm Nunc cell culture/Petri dishes (ThermoFisher, Catalog no. 168381)
10. Hemocytometer

### Anther isolation procedure

1. Collect fresh wheat heads and place on an RNase free plastic Petri dish under the dissection microscope.

- It is critical to clean all plasticware (Petri dishes, microscope slides and coverslips) used throughout the extraction procedure with absolute alcohol initially and then with RNase Away solution to prevent RNA degradation by RNase.

2. Excise individual florets from the spikelets and place them in a drop of 1X DPBS (~ 200 μL) to avoid drying due to heat from the light source.

- At this stage, a pool of florets collected from a single wheat head is distributed into multiple DPBS drops.

3. Carefully isolate anthers from individual wheat florets by removing them from the floret pools.

- The three anthers from individual florets are placed separately and care should be taken to avoid mixing of anthers from different florets or spikelets.

4. Place the three anthers isolated from a single wheat floret in 50 μL 1X DPBS solution on an RNase free petri dish.
5. Repeat above steps until 12–15 anthers are collected and place them in individual DPBS drops (each drop with 3 anthers from a floret).

- To obtain a population of 5000 meiocytes, at least 40 to 50 anthers of the right stage are required.
- Do not prolong the anther isolation procedure for more than an hour to avoid possible inconsistency in gene or protein expression patterns.

### Size sorting and staging of meiotic anthers

6. Place the isolated anthers from every single floret under the dissection scope fitted with an ocular micrometer and measure the length of anthers.

- The ocular micrometer has to be calibrated before size sorting. Place a stage micrometer (Cole-Parmer, Montreal, QC, Canada) on the stage and adjust with focus knob of the dissection microscope (with 10X objective) to position the ocular micrometer in such a way that both scales are completely parallel and each unit of the ocular micrometer is lined up with the units of the stage micrometer. Calibrate the ocular micrometer each time when the objective is changed.
- To determine the size of the anthers, measure the length of the longest locule from base to the tip in millimeters.

7. Sort anthers according to the length ranging from 0.7 to 1.2 mm.
8. For meiotic staging, remove one anther from each floret DPBS pool and squash it on a glass microscopic slide, stain it by adding 15–20 μL of 2% acetocarmine and place a glass coverslip over the squash.

- One of the three anthers from each floret is always sacrificed to confirm the meiotic stage of the anther. This quality control step prevents incorrect anthers to be pooled together for SAC isolation.

9. Using a light microscope, record the meiotic stage of the squashed anther and collect the remaining two anthers for SAC isolation.
10. Repeat steps 8 and 9 for the rest of the anther pools until enough meiocytes are collected for each meiotic stage.

### Isolation of intact SACs containing meiocytes

11. Take an RNase free plastic microscope slide, add 50 μL of 1X DPBS to the left of the slide and mark it as drop number one and make sure the drop forms a perfect dome shape.

- The use of plastic cover slips is recommended as it retains the perfect dome shape of the droplet of DPBS collection buffer.
- The dome shaped droplets create enough room for microdispenser manipulation, as the heavier non-sporogenous tissues from the anthers settle at the bottom and the meiocytes float within the droplet.

12. Using the dissection microscope, pool and transfer at least 10 anthers of the same meiosis stage to the DPBS drop.

- The pooled anthers should come from the set of size sorted and stage identified anthers in previous steps.

13. Using a dissection needle, gently make a nick at the narrow end of individual anther in the DPBS dome.

- Avoid the use of regular dissecting needles. A self-made dissecting needle, made by gluing an insect pin on a 3 mm thick bamboo skewer, can be used to tease apart the anthers and release SACs. This will prevent any excessive damage to the integrity of the SACs.

14. Place the dissection needle on the broad end of the nicked anther and gently roll the needle towards the narrow end to push the SACs out and release them from the locules of the anther.

- Due diligence is necessary to avoid breaking the SACs when squeezing the anthers from the broad end.

15. Repeat steps 13 and 14 for all 10 anthers placed in the DPBS drop. Add more 1X DPBS (50–150 μL) if necessary, and make sure the dome structure is retained throughout the extraction procedure.
16. Gently disrupt the base of the dome to release SACs sticking on to the surface of the dome. Place the microscope slide under an inverted microscope and view SACs under a 5X objective.

- Care should be taken to avoid excessive heating from the light source of the inverted microscope. In the present study, we removed the integral incandescent light source of the inverted microscope and replaced it with a table lamp fitted with a white LED light. This modification provided additional space during SAC collection and it also prevented drying of collection buffer due to excess heating from the integral light source.
- Make sure the SACs are free floating in the DPBS drop to make the isolation procedure efficient.
- Notably, SACs remain intact from the pre-meiotic stage until diakinesis (end of prophase-I) and start to disintegrate by the end of diakinesis.

17. Add another drop of 1X DPBS to the right side of the slide and mark it as drop number two.

- The two-step cleanup procedure ensures high purity of meiocytes with no contamination from non-sporogenous cells. Occasionally additional cleanup steps are required to obtain purified SACs devoid of somatic cells.

18. Using a 5 μL calibrated Drummond microdispenser, collect and transfer individual SACs from drop number one to two, observe under the microscope and remove contaminating debris using a microdispenser.

- By using a microdispenser attached to a capillary tube, this step leverages the advantages of both traditional micromanipulation and the CCM method and avoids mouth pipetting during the isolation procedure.

19. Transfer clean intact SACs from drop number two to a micro-centrifuge tube containing 100 μL of RNAlater solution.
20. Repeat above steps until a desired amount of SACs or meiocytes have been collected.

- In an hour, typical amounts of meiocytes collected differed per stage and were as follows: 500 to 700 (premeiotic G2), 1000 to 1200 (leptotene), 1000 to 1200 (zygotene), 1400 to 1500 (pachytene), 1500 (diplotene), 300 to 400 (diakinesis), 700 to 800 (metaphase I).
- The duration of meiosis in plants is a significant aspect in identifying and staging meiosis I anthers. MeioCapture was thus performed for a maximum time of 4 h (premeiotic), 3–4 h (leptotene), 3 h (zygotene), 2 h (pachytene), 0.5 h (diplotene), 0.5 h (diakinesis) and 1.5 h (metaphase I) per day to match the collection time with the respective meiotic duration and avoid mixed meiotic stages.

21. Collected SACs can be stored in RNAlater solution at 4 °C for up to 4 weeks before RNA extraction. If the isolated meiocytes were to be used for protein extraction, freezing the meiocytes in DPBS with liquid nitrogen and storing at − 80 °C is recommended.

### Meiocytes purity check

22. Using a wide bore pipette tip gently mix the SAC suspension collected from different meiotic stages (corresponding to specific anther lengths) to break the column structure and release the meiocytes into the solution.
23. Transfer 1 μL of meiocyte suspension to a 0.5 mL microfuge tube and add 1 μL of 2% Acetocarmine solution to the resuspended meiocytes solution. Mix it gently again by pipetting using a wide bore pipette tip.
24. Add the stained meiocytes on to a hemocytometer, and count and record the number of meiocytes present at each specific meiotic stage under a light microscope.

- Repeat steps 23 and 24 for 9 more times for each meiotic stage to a total of 10 replications. Calculate the average number of meiotic stages obtained from the 10 replications.

25. Calculate the percentage of individual meiotic stages observed in different SAC suspensions.

## Results

### Isolation of SACs

**Fig. 3:**
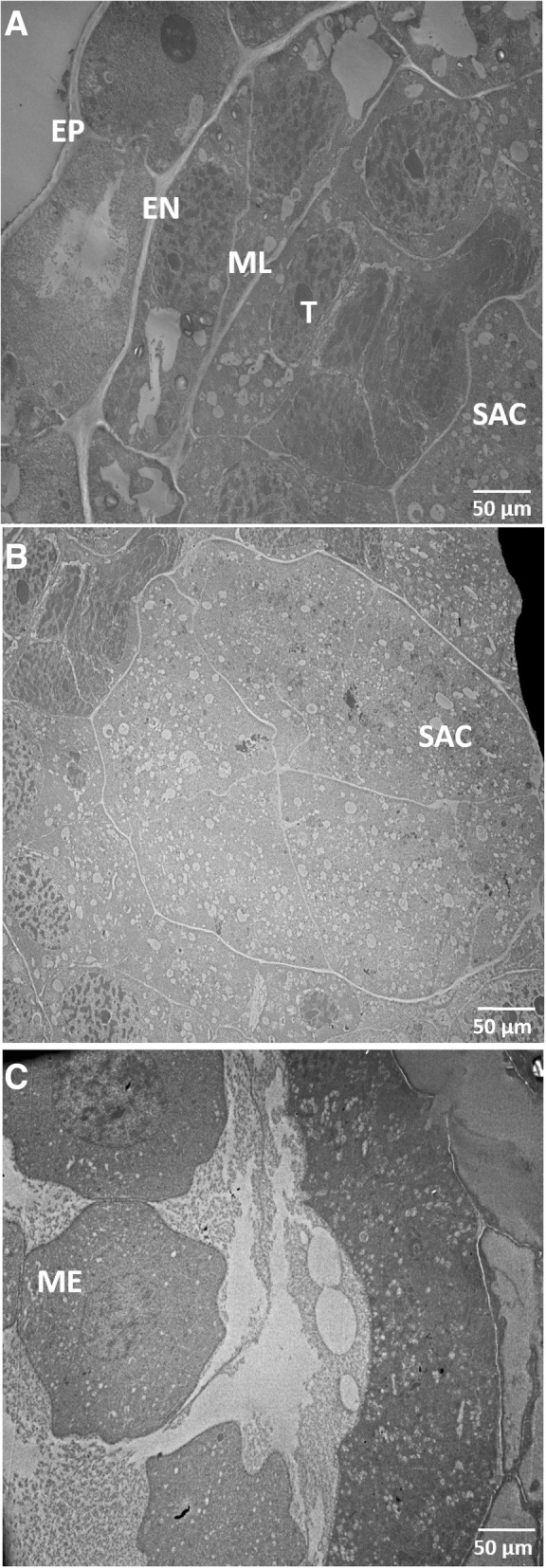
Transmission electron microscopic (TEM) images of anther locules in Chinese Spring wheat. **a** An ultra-thin section of 0.5 mm anther visualized by TEM. EP, epidermis; EN, endodermis; ML, middle layer; T, tapetum; SAC, sporogenous archesporial column. **b** Central region of the anther showing the transverse section of a SAC. **c** TEM image of an ultra-thin section of 1.4 mm anther showing disintegrated SAC resulting in dissociation of meiocytes (ME). **b**, **c** and **d** are in different magnification

**Fig. 4:**
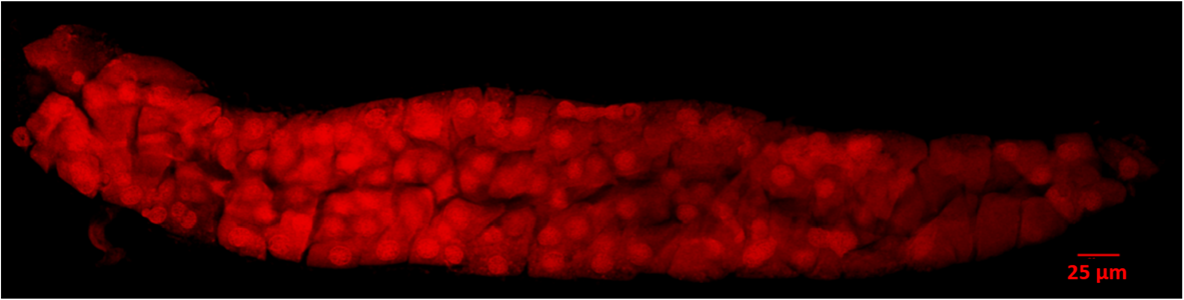
A sporogenous archesporial column (SAC) visualized by confocal microscopy. The pseudo-coloured image shows a maximum projection of a 14.7 μm thick Z-stack (20 slices)

**Fig. 5:**
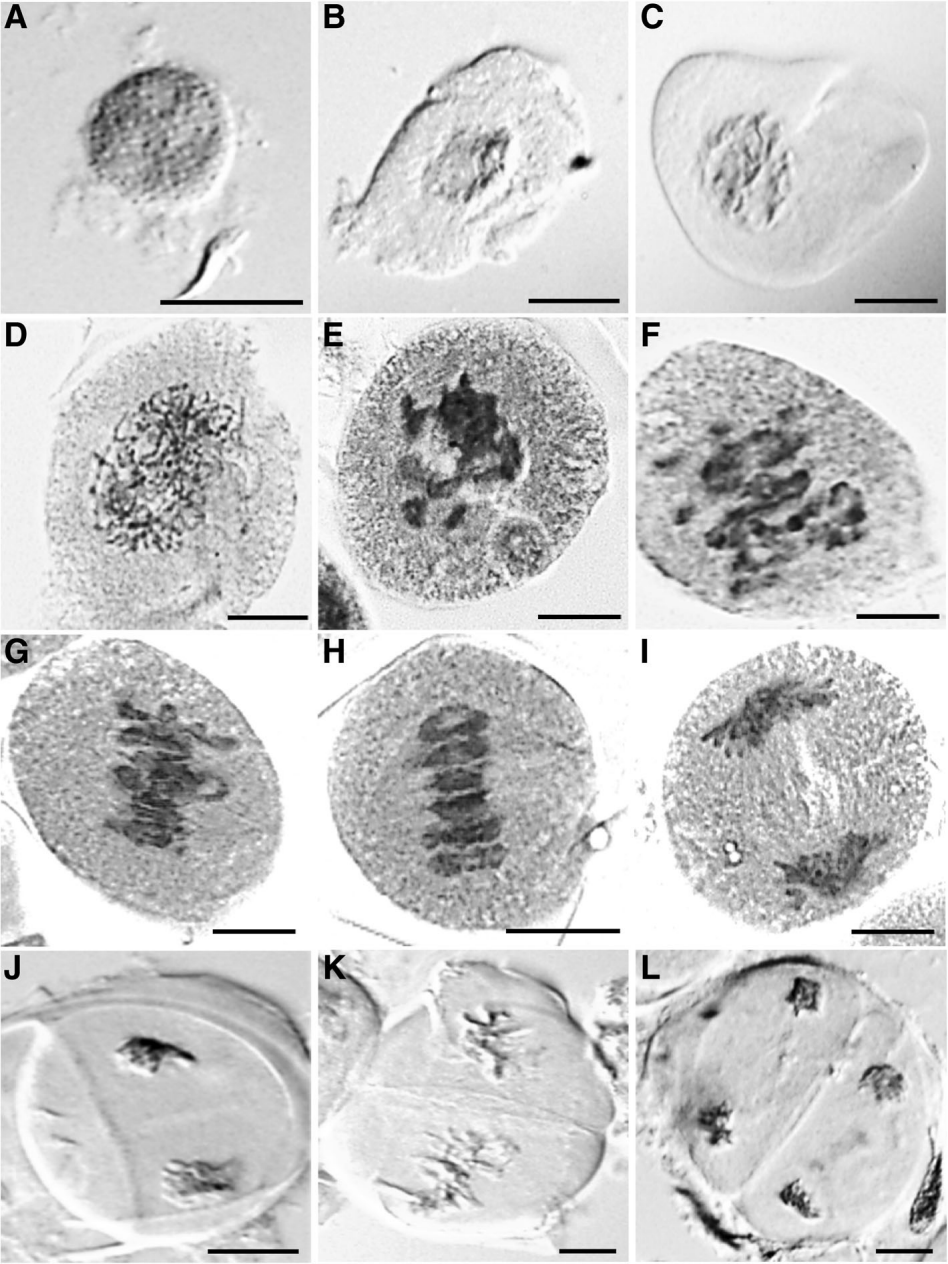
Light microscopic meiotic atlas of wheat showing the different stages of meiosis, **a** premeiotic G2 nuclei; **b** leptotene; **c** zygotene; **d** pachytene; **e** diplotene; **f** diakinesis; **g** early metaphase I; **h** metaphase I; **i** anaphase I; **j** telophase I; **k** metaphase II and (**l**) telophase II. The microscopic magnifications of the stages are different as the focus was to show chromosomal arrangements within the cells. Scale bar = 25 μm

SACs form a distinct layer in each locule and hence can be easily extruded out of the anther. Hence, our strategy to isolate uncontaminated pure meiocyte subpopulations involved extrusion of intact SACs, which can be achieved by creating a nick at the narrow end of individual anther and gently rolling a dissection needle from the broader tip of the anther towards the narrow end. This releases the SACs present in all four locules (for detailed procedures see Methods section). Transmission electron microscopy (TEM) images of anthers ranging from 0.5 to 1.4 mm in length revealed that the SAC retains its integrity throughout the prophase I process but eventually disintegrates as the meiocytes develop into microspores (Fig. 3, Additional file 1: Figure S1). For example, an intact SAC is present at the pre-meiotic stage (0.5 mm anther; Fig. 3a and b), but a disintegrated SAC with free floating meiocytes was observed after the completion of the meiosis-I process (1.4 mm anther; Fig. 3c). A confocal microscopy image of an intact SAC isolated using the described method is shown in Fig. 4. A light microscopic atlas of meiosis in CS genotype of wheat was developed using acetocarmine staining of meiocytes isolated using the MeioCapture method (Fig. 5).

### Anther size as a marker for meiotic stages

**Fig. 6:**
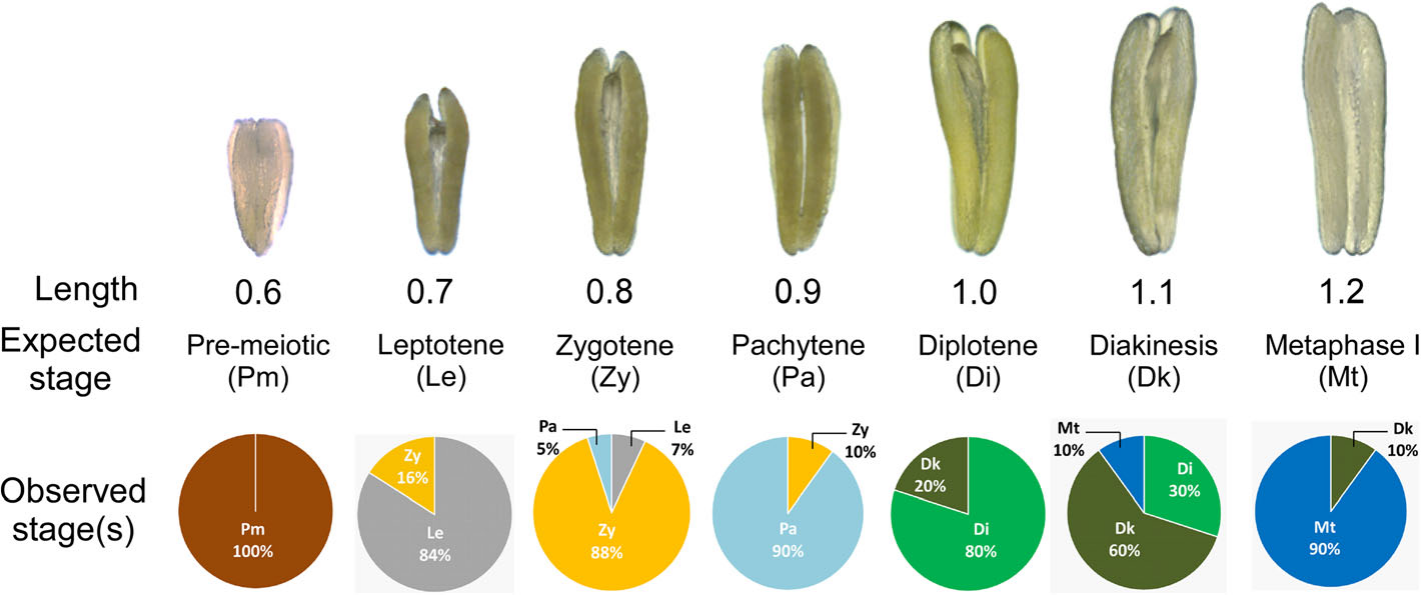
Correlation between length of anthers and meiotic stages in the Chinese Spring genotype of wheat and efficiency of MeioCapture method. Anthers from 0.6, 0.7, 0.8, 0.9, 1.0, 1.1 and 1.2 mm contained meiocytes predominantly at pre-meiotic, leptotene, zygotene, pachytene, diplotene, diakinesis and metaphase I stages respectively; anther length was measured using an ocular micrometer. The purity of meiocytes isolated from each sub-stage of prophase I of meiosis using MeioCapture is shown. For each extraction, the values are expressed as percentage of meiocytes occurring in unique or different stages of meiosis. The values are calculated from an average of 10 replications for each stage. Pm, pre-meiotic; Le, leptotene, Zy, zygotene; Pa, pachytene; Di, diplotene; Dk, diakinesis; Mt., metaphase I

Several previous studies have consistently shown a correlation between anther sizes and the meiotic stages in *T. aestivum* [9], *Zea mays* [35–38], *A. thaliana* [39] and *Arabidopsis arenosa* [40]. To verify such correlation, we carefully staged CS wheat meiocytes from multiple anthers of varying lengths. It was observed that in CS grown at 21 °C, anthers of 0.5 to 0.6, 0.7, 0.8, 0.9, 1.0, 1.1 and 1.2 mm length contained meiocytes predominantly in the pre-meiotic, leptotene, zygotene, pachytene, diplotene, diakinesis and metaphase I stages, respectively (Fig. 6), suggesting that anther size can be used as a reliable marker for meiotic staging in wheat under controlled environmental conditions.

### Efficiency of MeioCapture

Although the MeioCapture protocol is based on isolation of SACs, a few somatic cells were recovered during each extraction. To avoid contamination of meiocytes, a two step-cleaning procedure (see step 17 in the MeioCapture protocol) was implemented. MeioCapture was able to isolate meiocytes from each sub-stage of prophase-I at a high level of purity, but some cross-contamination from adjoining stages was evident at later sub-stages of prophase I (Fig. 6). While the pre-meiotic suspension was 100% pure with no contamination from other sub-stages, the leptotene suspension was 84% pure with the remaining cells progressed to the zygotene stage. Similarly, the zygotene, pachytene, and diplotene suspensions had slight cross-contamination from meiocytes from adjoining sub-stages (Fig. 6). The lower purity (60%) of meiocytes in diakinesis stage can be attributed to the short duration of this stage; it lasts only for 0.4 h (Table 1) and hence incurs contamination from adjoining stages (diplotene and metaphase I). The meiocytes purity check was performed using 10 replications of isolated meiocytes using MeioCapture (Fig. 6, Additional file 2: Table S1). Two other spring wheat genotypes, Stettler [41] and Fielder [42], also had similar meiotic stage-to-anther-size correlations for prophase I stages (Additional file 3: Table S2). The analysis of variance estimates showed no significant differences (Analysis of variance, *P* > 0.9) in the success rate in identifying the correct prophase I sub-stages based on anther size correlation between CS, Fielder and Stettler (Additional file 3: Table S2).

## Discussion

In this article, we describe an easy and reproducible method termed MeioCapture for simultaneous isolation of high purity male meiocytes progressing through various stages of meiosis. In contrast to previous procedures which involved collection of individual meiocytes, the MeioCapture protocol involves extrusion of intact meiotic columns, SACs (Fig. 4) containing pools of meiocytes. The complex anatomy of the anther makes accessing and isolation of meiocytes difficult. However, the meiotic and non-meiotic cell layers of the anther arise from different cell lineages [8], and hence meiocytes in the anther are formed as a distinct column of cells (SACs) that can be easily extruded without the contamination of somatic cells. The natural meiotic synchrony between anthers of the same floret and the correlation between anther size and meiotic stage (Fig. 6) offer huge advantage by reducing the time and effort required for simultaneous isolation of high purity subpopulations of prophase I meiocytes and ensure the reproducibility of the technique.

The environment and plant growth conditions are critical for reproducibility of the protocol. Similarly, the understanding of the duration of each sub-stage of meiosis is essential to minimize the cross-contamination during meiocyte isolation. The duration of meiosis in PMCs of wheat, rye and triticale have been studied in the past [43]. The whole meiotic process in wheat takes 24 h of which prophase I alone lasts for 16 h [43]. Leptotene is the longest prophase I sub-stage (10.4 h) followed by zygotene (3.4 h), pachytene (2.2 h), diplotene (0.6 h) and diakinesis (0.4 h). The variable duration of each sub-stage of prophase I has an impact on isolation of uncontaminated meiocytes. The frequency of cross-contamination increased significantly whenever the meiocyte collection procedure prolonged beyond the length of a particular sub-stage. The occurrence of multiple stages in an anther of particular length could also be due to the presence of developmental gradient along the anther axis. The base of the anther (broad end) contains SAC cells at early stages of meiosis and advance as they reach the tip of the anther (narrow end). However, it was found that this development gradient did not affect the synchrony of meiocytes when the meiotic stages were completed within 1 to 2 h [9]. Thus it is essential to isolate meiocytes quickly and efficiently within the duration of each meiotic stage or sub-stage being handled.

The MeioCapture procedure has been successfully used to isolate stage-specific populations of meiocytes in multiple genotypes of wheat. This method is predicted to be applicable to other crop species provided prior knowledge of the meiotic synchrony between anthers of the same flower as well as anther size and meiotic stage correlation is available. The protocol provides an essential technique for high-resolution omics studies needed to understand the molecular control of meiotic commitment and progression.

## Conclusion

The high quality and quantity of meiocytes obtained by MeioCapture method will facilitate future genetic, cytogenomic and proteomic analysis of meiosis in plants. Although cytogenetics has answered many key questions about meiosis, the genetic basis of chromosome pairing and homoeologous recombination is still not fully understood in many polyploid crop species. With the establishment of this essential technique for meiocyte isolation and the recent availability of genomics resources, wheat can provide a polyploid model for high-resolution transcriptomic and proteomic studies needed to understand the molecular control of chromosome pairing and recombination.

## Additional files

Additional file 1: **Figure S1.** Transmission electron microscopy images of ultra-thin sections of Chinese Spring wheat anthers varying in length from 0.5 to 1.4 mm. Scale bar = 50 μm. (PDF 570 kb)

Additional file 2: **Table S1.** Purity analysis of meiocytes isolated from Chinese Spring using MeioCapture protocol. Data was collected from at least 10 independent replicates for each meiotic stage. The numbers within each replication represent the number of meiocytes present in 1 μL of meiocyte extract. (XLSX 13 kb)

Additional file 3: **Table S2.** Purity analysis of meiocytes isolated from Stettler and Fielder using MeioCapture protocol. Data was collected from at least 3 independent replicates for each meiotic stage. The numbers within each replication represent the number of meiocytes present in 1 μL of meiocyte extract. The analysis of variance estimates showed no significant differences in success rate in identifying the correct prophase I sub-stages between CS, Fielder and Stettler. (XLSX 15 kb)

## A**bbreviations**

CCM: Capillary collection of meiocytes
CS: Chinese Spring
DPBS: Dulbecco’s phosphate-buffered saline
EN: Endodermis
EP: Epidermis
FACS: Fluorescence activated cell sorting
INTACT: Isolation of nuclei tagged in specific cell types
LCM: Laser capture microdissection
ME: Meiocytes
ML: Middle layer
NaCac: Sodium Cacodylate
PMC: Pollen mother cell
PPFD: Photosynthetic photon flux density
SAC: Sporogenous archesporial column
T: Tapetum
TEM: Transmission electron microscopy
