# Correction to: MeioCapture: an efficient method for staging and isolation of meiocytes in the prophase I sub-stages of meiosis in wheat

**DOI:** 10.1186/s12870-019-1780-4

**Published:** 2019-05-02

**Authors:** Arun S. K. Shunmugam, Venkatesh Bollina, Stefanie Dukowic-Schulze, Pankaj K. Bhowmik, Chris Ambrose, James D. Higgins, Curtis Pozniak, Andrew G. Sharpe, Kevin Rozwadowski, Sateesh Kagale

**Affiliations:** 1 National Research Council Canada, Saskatoon, SK Canada; 2 Department of Horticultural Science, University of Minnesota, St. Paul, MN USA; 3 Department of Biology, University of Saskatchewan, Saskatoon, SK Canada; 4 Department of Genetics and Genome Biology, University of Leicester, Leicester, UK; 5 Department of Plant Sciences, University of Saskatchewan, Saskatoon, Canada; 6 Global Institute for Food Security, University of Saskatchewan, Saskatoon, Canada; 7 Agriculture and Agri-Food Canada, Saskatoon, SK Canada

**Correction to: BMC Plant Biol (2018) 18:293**

**https://doi.org/10.1186/s12870-018-1514-z**

Following publication of the original article [1], a reader spotted an incorrect citation of the reference 14 [2] in the ‘Background’. The male meiocyte isolation work described in this article [2] was carried out in rice and not in Brassica as originally stated in the ‘Background’ [1]. Thus, the following amendment to the Background section should be noted:

Original sentence:

“Several methods have been previously described or proposed for the isolation of male meiocytes [6], such as micromanipulation (Plumbago [10, 11], Nicotiana [12], Brassica [13, 14], Arabidopsis [15, 16, 17] and sunflower [18]), capillary collection of meiocytes (CCM) (Arabidopsis [19, 20] and maize [21, 22]), laser capture microdissection (LCM) in rice [23, 24, 25, 26], Percoll gradient separation (Arabidopsis [27, 28], rice [29] and Brassica [30]) and isolation of nuclei tagged in specific cell types (INTACT) in Arabidopsis [31].”

Corrected sentence:

“Several methods have been previously described or proposed for the isolation of male meiocytes [6], such as micromanipulation (Plumbago [10, 11], Nicotiana [12], Brassica [13], rice [14], Arabidopsis [15, 16, 17] and sunflower [18]), capillary collection of meiocytes (CCM) (Arabidopsis [19, 20] and maize [21, 22]), laser capture microdissection (LCM) in rice [23, 24, 25, 26], Percoll gradient separation (Arabidopsis [27, 28], rice [29] and Brassica [30]) and isolation of nuclei tagged in specific cell types (INTACT) in Arabidopsis [31].”.
