# Supplementary material for: MeioCapture: an efficient method for staging and isolation of meiocytes in the prophase I sub-stages of meiosis in wheat
Source: BMC Plant Biol. 2018 Nov 21;18:293. doi: 10.1186/s12870-018-1514-z (PMC6249822; doi:10.1186/s12870-018-1514-z)
Supplement: Supplementary file 1 — Figure S1. Transmission electron microscopy images of ultra-thin sections of Chinese Spring wheat anthers varying in length from 0.5 to 1.4 mm. Scale bar = 50 μm. (PDF 570 kb) [file 12870_2018_1514_MOESM1_ESM.pdf]

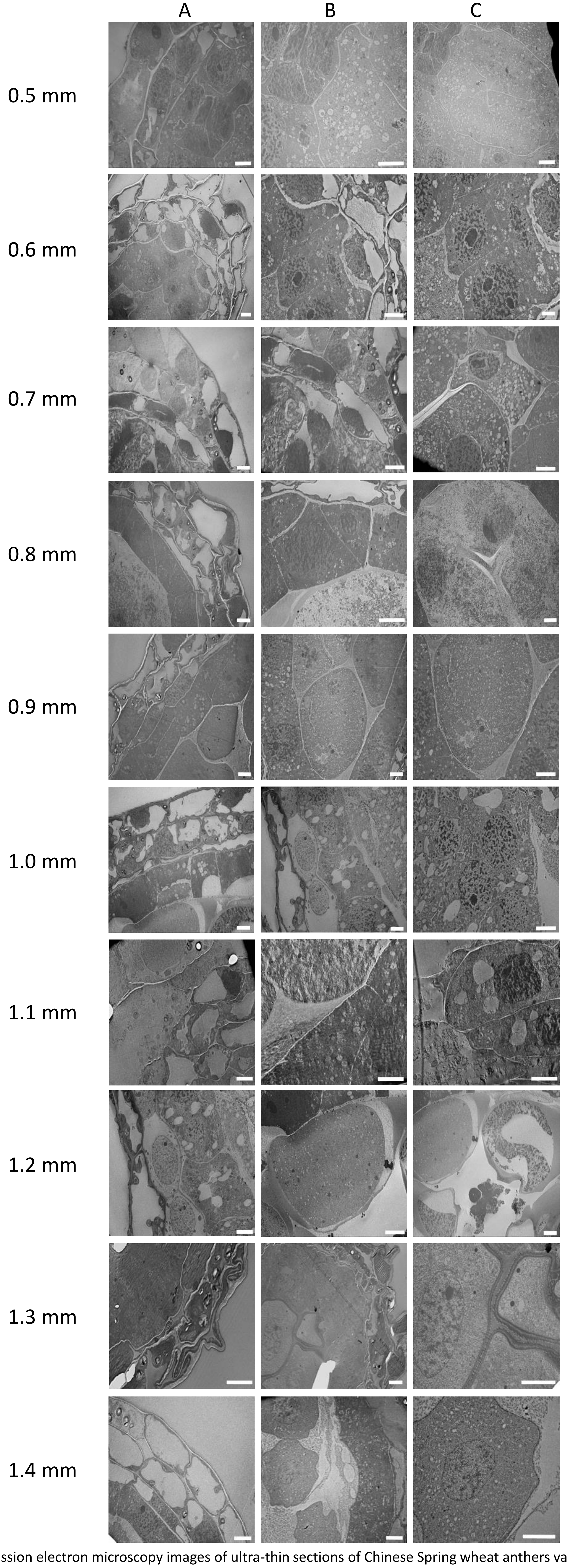

Figure S1. Transmission electron microscopy images of ultra-thin sections of Chinese Spring wheat anthers varying in length from 0.5 to 1.4 mm (A) Cross section showing the multiple layers of anther tissue (B) Tapetal and meiotic cells are shown for comparison and (C) **M**eiotic nuclei of the corresponding anthers are shown wherever possible. The images are captured at different magnifications to show changes in anther anatomy throughout the meiotic progression. Scale bar = 50 µm.
